# The glycoside hydrolase gene family profile and microbial function of *Debaryomyces hansenii* Y4 during South-road dark tea fermentation

**DOI:** 10.3389/fmicb.2023.1229251

**Published:** 2023-07-12

**Authors:** Yao Zou, Minqiang Liu, Yuqing Lai, Xuyi Liu, Xian Li, Yimiao Li, Qian Tang, Wei Xu

**Affiliations:** ^1^Department of Tea Science, College of Horticulture, Sichuan Agricultural University, Chengdu, China; ^2^Tea Refining and Innovation Key Laboratory of Sichuan Province, Chengdu, China

**Keywords:** *Debaryomyces hansenii* Y4, Sichuan South-road dark tea, glycoside hydrolase gene family, fermentation, quality

## Abstract

Microbes are crucial to the quality formation of Sichuan South-road Dark Tea (SSDT) during pile-fermentation, but their mechanism of action has not yet been elucidated. Here, the glycoside hydrolase (GH) gene family and microbial function of *Debaryomyces hansenii* Y4 during solid-state fermentation were analyzed, and the results showed that many *GH* genes being distributed in comparatively abundant GH17, GH18, GH76, GH31, GH47, and GH2 were discovered in *D. hansenii*. They encoded beta-galactosidase, alpha-D-galactoside galactohydrolase, alpha-xylosidase, mannosidase, etc., and most of the GHs were located in the exocellular space and participated in the degradation of polysaccharides and oligosaccharides. *D. hansenii* Y4 could develop the mellow mouthfeel and “reddish brown” factors of SSDT via increasing the levels of water extracts, soluble sugars and amino acids but decreasing the tea polyphenols and caffeine levels, combined with altering the levels of thearubiins and brown index. It may facilitate the isomerization between epicatechin gallate and catechin gallate. Moreover, the expression levels of *DEHA2G24860g* (Beta-galactosidase gene) and *DEHA2G08602g* (Mannan endo-1,6-alpha-mannosidase DFG5 gene) were sharply up-regulated in fermentative anaphase, and they were significantly and negatively correlated with epicatechin content, especially, the expression of *DEHA2G08602g* was significantly and negatively correlated with catechin gallate level. It was hypothesized that *D. hansenii* Y4 is likely to be an important functional microbe targeting carbohydrate destruction and catechin transformation during SSDT pile-fermentation, with *DEHA2G08602g* as a key thermotolerant functional gene.

## Introduction

**Figure 1:**
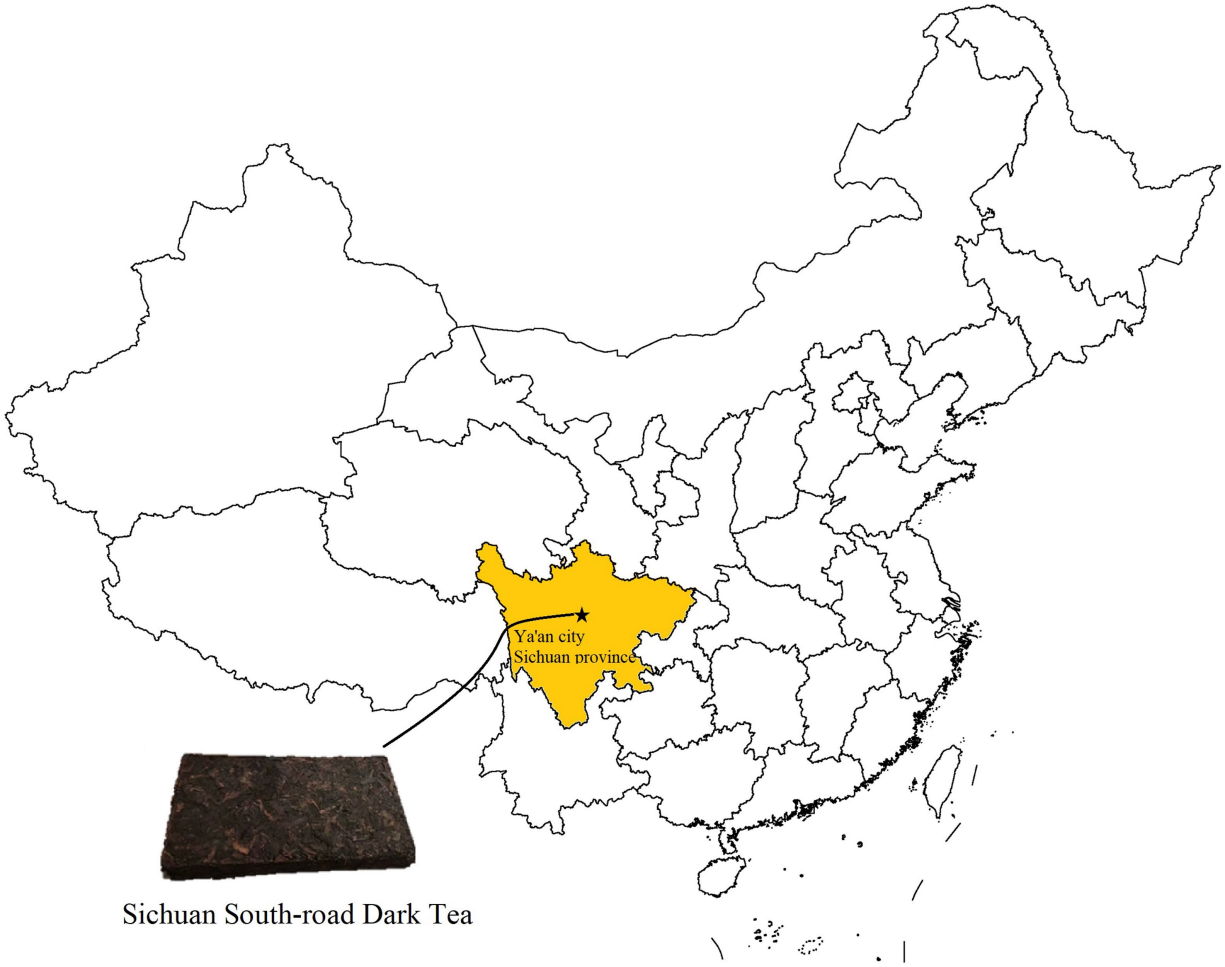
Sichuan South-road Dark Tea. The yellow area reflects Sichuan province and the pentagram means Ya’an city, the origin of SSDT.

South-road Dark Tea (SSDT) is a well-known health beverage in China, it is produced in Ya’ an City, Sichuan Province (Figure 1), and characterized by the sensory qualities of brick shape, reddish-brown appearance, mellow mouthfeel, and aged and pure aroma (Zou et al., 2022). Pile-fermentation, a spontaneous fermentation stimulated by environmental microorganisms in workshop, is the key procedure responsible for SSDT quality formation, during which microbial metabolism, extracellular enzyme activities and natural oxidation, etc. accelerate the transformation of complex compounds, produce various secondary metabolites, ultimately contribute to the unique flavor of dark tea (Zhang et al., 2016). Recently, some of the functional microorganisms involved in the distinctive flavor formation of dark tea have been identified, for example, *Aspergillus*, *Debaryomyces*, and *Lichtheimia* were confirmed to be the primary beneficial agents of Pu-erh tea during fermentation (Li et al., 2018; Ma et al., 2021), while *Aspergillus* and *Debaryomyces* also play an important role in the volatile metabolism of Fuzhuan tea during production (Li M. Y. et al., 2020). Furthermore, *Aspergillus niger* M10 could significantly influence the transformation of key quality components in SSDT during fermentation via the expression of *GH* genes (Zou et al., 2022, 2023). It seems that the functional microbes are very important in the production of dark tea.

The raw materials for processing SSDT are the mature leaves and branches of tea plant, which are rich in cellulose, hemicellulose and other polysaccharides. Usually, cellulose and hemicellulose are cross-linked by lignin and pectin, etc. to form plant cell walls (Burton et al., 2010). During pile-fermentation, tea leaf cell wall would be destroyed by the superposed effects of hygrothermal fermentation environment and microbial action, which promotes the conversion of chemical components and finally benefits dark tea quality (Wang et al., 2011). Published literature suggests that various carbohydrate-active enzymes (CAZymes) are involved in the degradation of leaf cell walls (Zou et al., 2023), of which GHs are the major modules responsible for hydrolyzing glycosidic linkages between carbohydrates or a carbohydrate-aglycone moiety (Tingley et al., 2021), thus playing a pivotal role in the degradation of complex carbohydrates. Generally, GHs contain different families, and members of the same family share more than 30% sequence similarity in primary structures (Henrissat, 1991), therefore present the similar functions, for instance, members of GH48 and GH6 mainly participate in destruction of cellulose, while members belonging to GH10, GH11, GH39, and GH43, etc. are primarily responsible for decomposing hemicellulose (Singh et al., 2019), some members of GH3 and GH1 could improve tea flavor by cleaving glycoside aroma precursors (Zhou et al., 2017; Xiang et al., 2020). Additionally, the researchers discovered that the expression of some *GH* genes, such as *NI_1_1714074* and *ANI_1_2704024*, may be significantly related to the degradation of polysaccharides (Zou et al., 2023). Overall, it is speculated that GHs probably play a critical role in dark tea quality formation during pile-fermentation.

*Debaryomyces* is a functional genus for dark tea production (Li et al., 2018), but the details of its species information and action mechanism during pile-fermentation are still obscure. We have ever isolated *Debaryomyces hansenii* Y4 (*D. hansenii* Y4) from stacked SSDT during pile-fermentation. Given the role of *D. hansenii* in the chemical transformation during black tea fermentation (Pasha and Reddy, 2005), the object of this work is to explore the function of *D. hansenii* Y4 during SSDT fermentation via analyzing microbial *GH* gene and detecting chemical components and color parameters of SSDT. The results may provide a theoretical basis for further research on the potential functions of *GH* genes, and elucidate the mechanism of organoleptic quality development of SSDT during pile-fermentation to some extent.

## Materials and methods

### *GH* gene family analysis

The genome sequence data of *D. hansenii CBS 767* and the sequence data of GH family modules were separately downloaded from the NCBI^1^ and Pfam^2^ databases. Utilizing BLASTP program to identify the amino acid sequences of GHs in *D. hansenii* based on the HMMER3.0 profile of the GHs domain (Supplementary Table S1). Amino acid length, molecular weights and theoretical isoelectric point (pI) of each protein were predicted with ExPASy^3^ Subcellular localization was predicated using WOLF PSORT (https://wolfpsort.hgc.jp/; Gasteiger et al., 2003). Using MEGA v.7.0 combined with neighbor joining (N-J) method to construct the phylogenetic tree, and whose reliability was tested with a bootstrap value of 1,000 (Kumar et al., 2008). Moreover, MapChart (Version 2.1) was employed to present the chromosomal distribution of *GH* genes (Li Q. et al., 2020), and their structure was generated by MapInspect.^4^ Simultaneously, protein sequence motif analysis was performed with MEME (Bailey et al., 2009), the conserved motif size was set as 6–50 amino acids and the maximum number of structural domains outputted was 15, motif structure was displayed using TBtools software (Chen et al., 2020).

### Solid-state fermentation

*Debaryomyces hansenii* Y4 (NCBI ID: OQ975970; Supplementary Figure S1) isolated from SSDT was activated and suspended in distilled water to a final concentration of 10^6^cfu/ml. Subsequently, Maozhuang teas (the raw materials of SSDT) were lightly crushed and their water content was adjusted to 30%, then placed in triangular flasks with air-vent capping (35 g per flask) and sterilized by autoclave. Part of the sterilized samples were inoculated with *D. hansenii* Y4 suspension (1 ml/flask), while the rest were inoculated with equivalent volumes of sterile distilled water as control (CK). After thorough mixing, all samples were fermented for 20 days at 55°C in a constant temperature and humidity incubator (GZ-120-HSH, Guangzhi, China).

Experiments were performed in triplicate and samples were collected every 2 days. Part of the samples were used to analyze the chemical components and color parameters, while the rest were stored in a −80°C refrigerator for quantitative real-time PCR (qRT-PCR) analysis.

### Chemical analysis and color parameters detection

According to the method described by China National Standard GB/T8305-2013, GB/T8313-2008 and GB/T8314-2013, water extract content (WE), tea polyphenols (TPs) and amino acid (AA) in tea samples were quantified, respectively. Furthermore, the levels of catechin monomers and caffeine (Caf) were determined following GB/T 8313–2018 with some modifications by Tan et al. (2021), while water-soluble sugar (SS) were detected by the method of Zou (2014). The contents of theaflavin (TF), thearubigin (TR) and theabrownin (TB) were, respectively, measured using spectrophotometric methods (Huang, 1997). Additionally, the CIELab parameters of dried tea and tea liquor were separately investigated as described by Zou et al. (2020), and the derivative parameters of tea pigments and CIELab parameters were calculated according to the formulas listed in Supplementary Table S2.

### RNA extraction, cDNA synthesis, and qRT-PCR analysis

*Debaryomyces hansenii* Y4 in different fermentation samples were, respectively, collected by centrifugation and differential centrifugation, and then ground using liquid nitrogen. The M5 plant RNeasy Complex Mini Kit (Mei5bio, Beijing, China) was used to extract their RNA, and after checking the concentration and integrity of RNA, the M5 super plus qPCR RT Kit (Mei5bio, Beijing, China) was utilized to synthesize the cDNA, followed by qRT-PCR using the CFX96™ Real-time PCR System (Bio-Rad, California, USA). All experiments were conducted in triplicate, and the 26S rRNA was used as a reference gene to normalize gene expression. The relative expression level of *GH* gene was calculated with the 2^−ΔΔCT^ method (Pfaffl, 2001). The primers used in this work were designed using Primer Premier 5.0 software and their details are presented in Supplementary Table S3.

### Statistical analysis

Pearson correlation analysis between *GH* gene expression level and chemical component content was performed using SPSS 22.0 (SPSS, Inc., Chicago, IL), and one-way ANOVA with LSD multiple comparison test was also conducted. Orthogonal partial least squares discriminant analysis (OPLS-DA) was carried out utilizing SIMCA 14.1 (Umetrics Corporation, Umeå, Sweden).

## Results

### Analysis of *GH* gene family in *Debaryomyces hansenii*

**Figure 2:**
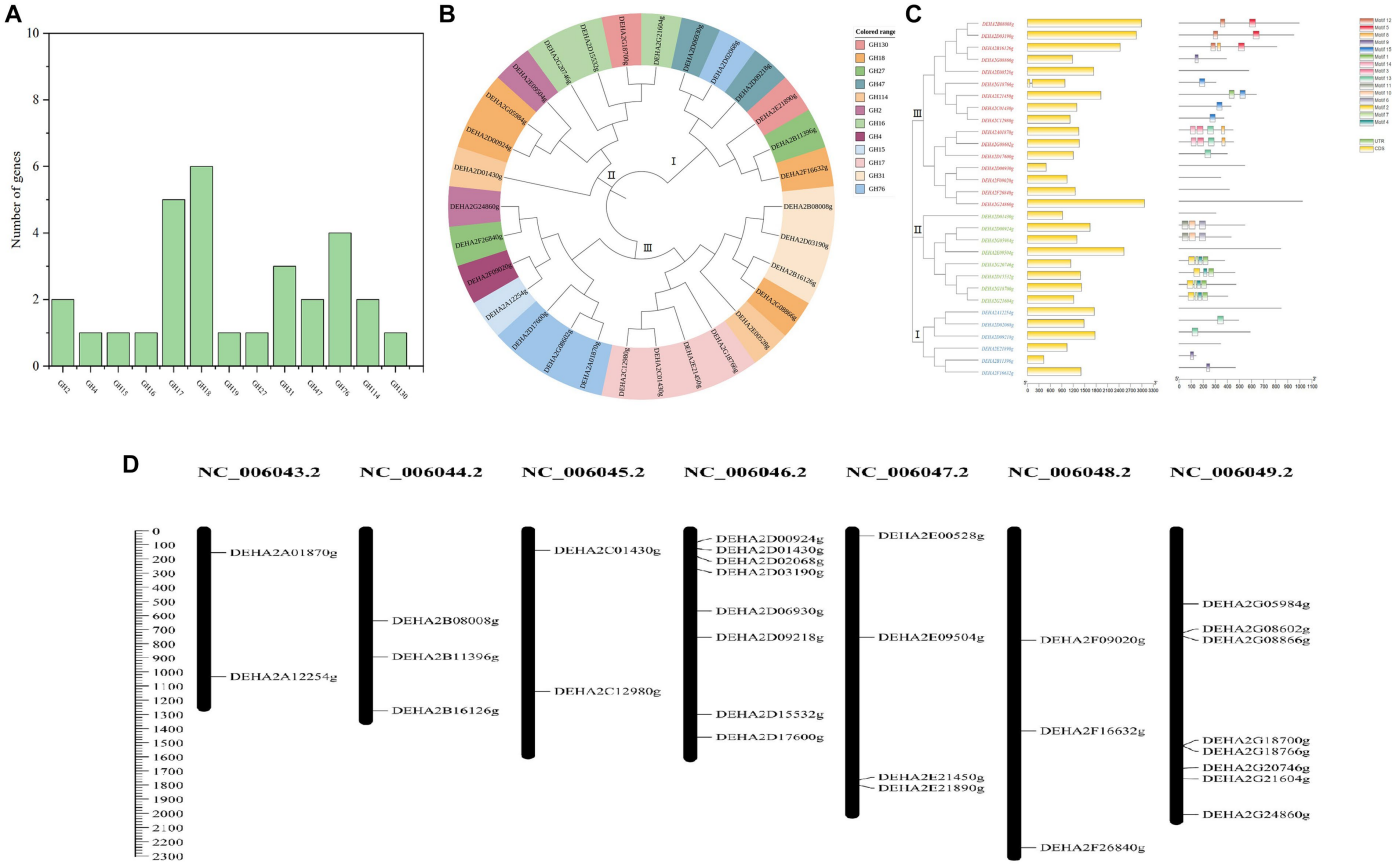
Analysis of *GH* gene family in *D. hansenii.* **(A)** GH family members in *D. hansenii.* **(B)** Phylogenetic analysis of GHs; the different colors indicate different GH families. **(C)** Exon-intron structure and conserved motifs analysis of *GH* genes in *D. hansenii*; boxes and lines, respectively, represent exons and introns in “exon-intron structure,” different colored boxes represent different motifs. **(D)** Chromosomal distribution of *GH* genes; chromosome numbers are showed at the top of each bar, and the size of chromosomes are reflected by the scale bar on the left.

A total of 30 proteins with typical GH domains were identified in *D. hansenii*, they were distributed in 13 GH families, with members of GH17, GH18, GH76, GH31, GH47 and GH2 being more abundant (Figure 2A). The phylogenetic analysis indicated that these protein sequences were classified into 3 distinct groups, among that group I was the smallest group with 6 members and contained all the genes encoding the members of GH 47; group II contained all the genes encoding the members of GH16; while group III formed the largest group with 16 members, and contained all the genes encoding the members of GH17, GH4, GH15 and GH31(Figure 2B). Obviously, the proteins belonging to the same GH family were clustered together, whereas, the conserved motifs of DEHA2G24860g (beta-galactosidase, GH2) and DEHA2F26840g (alpha-D-galactoside galactohydrolase, GH27) seem to be highly homologous since they were branched together. Moreover, all the genes detected contained exons, but only *DEHA2G18766g* (Glucan 1,3-beta-glucosidase gene) had an intron. In order to elucidate the composition and diversity of motifs in these protein sequences, MEME Suite was utilized to search for protein motifs, finally identifying 15 distinct motifs ranging from 20 to 50 amino acids in length. Noticeably, motifs 9 and 13 were mainly detected in group I, while motifs 1, 2, 4, 6, 7, 10 and 11 were primarily identified in group II, and motifs 3, 5, 8, 9, 12, 13, 14 and 15 were principally obtained in group III, nevertheless, motifs were absent in DEHA2E00528g, DEHA2D06930g, DEHA2F09020g, DEHA2F26840g, DEHA2G24860g, DEHA2D01430g, DEHA2E09504g, DEHA2A12254g, and DEHA2E21890g (Figure 2C; Supplementary Table S4). Furthermore, all genes were found unevenly distributed on 6 chromosomes, with more genes located on NC_006046.2 and NC_006049.2, while NC_006043.2 and NC_006045.2 had only two genes. Simultaneously, the clustering phenomenon was confirmed in NC_006049.2 as *DEHA2G18700g* and *DEHA2G18766g* were accumulated on the same region of this chromosome (Figure 2D).

Additionally, 18 genes encoding glycoside hydrolase were subjected to further analysis, and the GHs comprising of beta- galactosidase, alpha-D-galactoside galactohydrolase, alpha-xylosidase, mannosidase and so on were confirmed. These GHs presented amino acid lengths ranging from 306 to 1,022, molecular mass ranging from 33.75 to 119.03 kDa, and PI between 3.87 and 8.6, besides, most of the GHs were located in the extracellular space, except for DEHA2G24860g and DEHA2F16632g located in the cytoplasm, DEHA2E09504g, DEHA2G08866g and DEHA2E00528g located in the nucleus, and DEHA2G20746g located in the endoplasmic reticulum (Table 1).

**Table 1:** Basic information of GH family members in *Debaryomyces hansenii.*

Gene names	Gene description	Accession number	GH family	Amino acids	Molecular weight/Da	Theoretical point	Unstable coefficient	Lipid solubility index	WoLF PSORT
*DEHA2G24860g*	Beta- galactosidase	XP_462623.1	GH2	1,022	119034.8	5.47	36.21	79.69	cyto
*DEHA2E09504g*	Mannosidase	XP_459719.1	GH2	843	97184.79	5.41	32.4	80.43	nucl
*DEHA2A12254g*	Glucoamylase	XP_002770029.1	GH15	545	62525.25	4.97	38.48	38.48	extr
*DEHA2G21604g*	Putative glycosidase of the cell wall	XP_462482.1	GH16	404	42289.55	4.32	46.69	59.21	extr
*DEHA2G18700g*	Putative glycosidase of the cell wall	XP_462353.1	GH16	472	49563.01	4.27	61.74	53.98	extr
*DEHA2G20746g*	Putative glycosidase of the cell wall	XP_462444.1	GH16	378	42673.56	4.94	32.97	76.08	E.R
*DEHA2G18766g*	Endo-beta-1 3-glucanase	XP_462355.1	GH17	306	33752.24	4.27	19.93	73.33	extr
*DEHA2C12980g*	Cell wall protein with similarity to glucanases	XP_458240.1	GH17	372	38980.85	5.06	25.55	71.29	extr
*DEHA2G08866g*	Chitinase	XP_461932.1	GH18	393	44573.55	6.56	37.68	73.92	nucl
*DEHA2F16632g*	Sporulation-specific chitinase	XP_461083.1	GH18	467	51454.29	8.67	26.3	66.64	cyto
*DEHA2D00924g*	Endochitinase	XP_458510.1	GH18	546	57380.75	3.87	31.7	78.77	extr
*DEHA2F26840g*	Alpha-D-galactoside galactohydrolase	XP_461506.1	GH27	417	46835.34	4.58	27.4	78.85	extr
*DEHA2B16126g*	Alpha-xylosidase	XP_457652.1	GH31	951	105819.6	4.64	40.86	77.5	extr
*DEHA2D03190g*	Glucoamylase	XP_458606.1	GH31	590	67544.58	5.36	30.35	73.52	extr
*DEHA2D09218g*	Mannosyl- oligosaccharide 1 2-alpha-mannosidase	XP_458865.1	GH47	590	67544.58	5.36	30.35	75.19	extr
*DEHA2A01870g*	Mannan endo-1,6-alpha-mannosidase DCW1	XP_456419.1	GH76	448	49977.85	4.48	25.48	76.65	extr
*DEHA2G08602g*	Mannan endo-1,6-alpha-mannosidase DFG5	XP_461921.1	GH76	452	50207.86	4.4	34.67	71.24	extr
*DEHA2E00528g*	Maltase	XP_459350.1	GH114	578	66747.65	4.85	37.4	74	nucl

nucl, nucleus; cyto, cytoplasm; extr, extracellular space; golg, golgi apparatus; E.R, endoplasmic reticulum.

### Effect of *Debaryomyces hansenii* Y4 on taste-active ingredients of SSDT during fermentation

**Figure 3:**
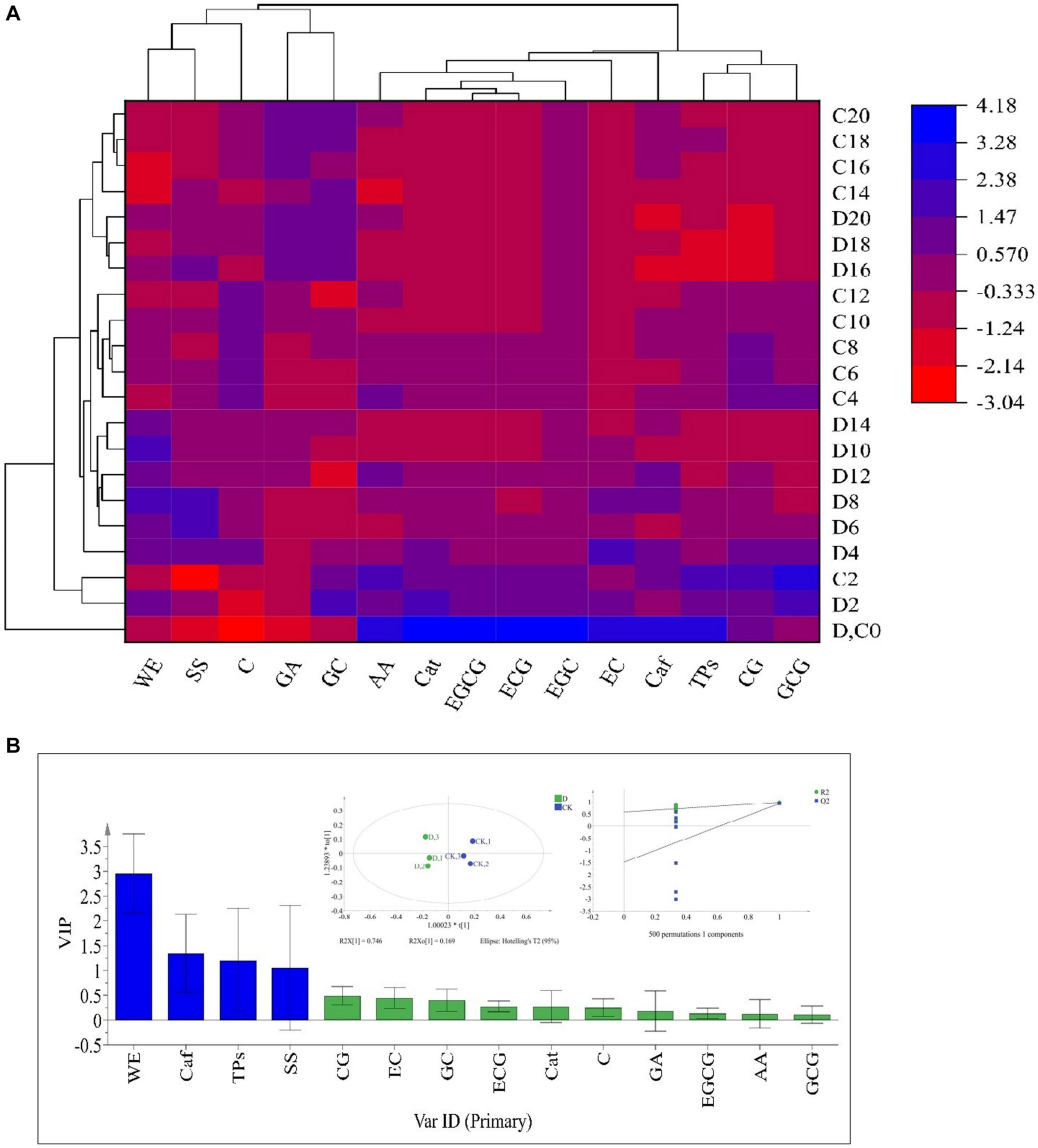
Analysis of the primary taste-active components of tea during solid-state fermentation. **(A)** heatmap of chemical ingredients; the row represents the samples at different fermentation times, C before the number represents the samples of CK, while D reflects the samples fermented by *D. hansenii* Y4, the column represents the taste-active components, the red and blue colors indicate their levels. **(B)** Orthogonal partial least square discriminant analysis (OPLS-DA) and determination of the differential chemical components.

The taste-active components levels of SSDT were apparently altered by solid-state fermentation. During fermentation, all the samples were clearly clustered into 4 groups: raw materials (0d), the 2d samples, the fermentative prophase and metaphase samples, and the fermentative anaphase samples. After fermentation, the levels of ECG, WE, GC and C in *D. hansenii* Y4 sample were significantly enhanced by 35.92,11.44, 11.8 and 14.11% (*p*<0.05), while SS and AA were increased by 8.73 and 2.93% (*p*>0.05), respectively, compared to CK. However, *D. hansenii* Y4 significantly decreased the contents of CG, EC, Caf and TPs in SSDT by 62.13, 25.29, 23.03 and 15.42%, respectively, as compared with that of CK (*p <* 0.05; Figure 3A). At the end of fermentation, although CK and *D. hansenii* Y4 samples were grouped together, but they were distinguishable from each other as shown in Figure 3B (OPLS-DA model: R^2^Y = 0.915, Q^2^ = 0.929; cross-validation with 500 permutation tests: intercepts of R^2^ = 0.579, Q^2^ = -0.148). Moreover, the differential chemical components between CK and *D. hansenii* Y4 samples after fermentation were observed with the criterion of VIP > 1 and *p* < 0.05, confirming that WE, Caf, TPs and SS were the differential taste-active chemical components between them (Figure 3B). The above results suggested that *D. hansenii* Y4 could significantly improve the mellow mouthfeel of SSDT by increasing the levels of thickness and sweetness-components but decreasing the contents of bitterness and astringency-compounds, and also altering the levels of catechins monomers.

### Effect of *Debaryomyces hansenii* Y4 on color parameters of SSDT during fermentation

**Figure 4:**
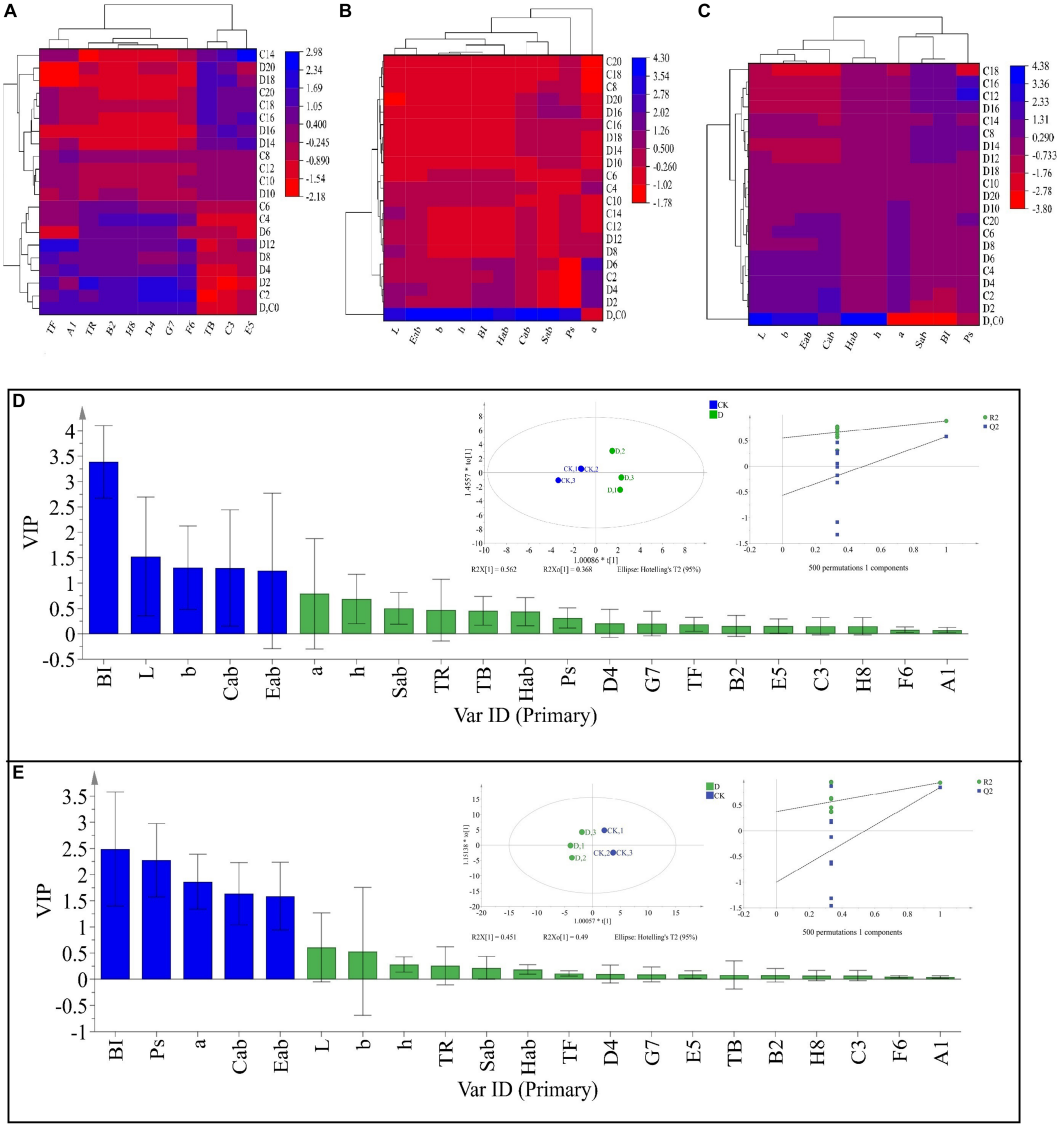
Analysis of the tea color parameters during solid-state fermentation. **(A)** heatmap of tea pigments and their derived parameters; the row represents the samples at different fermentation times, C before the number represents the samples of CK, while D reflects the samples fermented by *D. hansenii* Y4, the column represents the tea pigments and their derived parameters, A1 = TF/(TF + TR + TB), B2 = TR/(TF + TR + TB), C3 = TB/(TF + TR + TB), D4 = TR/TB, E5 = TF/TR, F6 = TF/TB, G7 = (TF + TR)/TB, H8 = (TF + TR)/(TF + TR + TB), the red and blue colors denote their levels. **(B)** Changes in CIELab parameters and their derived parameters of dried tea; the row and column represent the samples at different fermentation times and CIELab parameters, respectively. **(C)** Changes in CIELab parameters and their derived parameters of tea liquor; the row and column represent the samples at different fermentation times and CIELab parameters, respectively. **(D)** Orthogonal partial least square discriminant analysis (OPLS-DA) of color parameters of dried tea and determination of its differential color parameters. **(E)** Orthogonal partial least square discriminant analysis (OPLS-DA) of color parameters of tea liquor and determination of its differential color parameters.

Tea pigments are the major parameters affecting SSDT color, in this work *D. hansenii* Y4 was discovered to significantly decrease the levels of TF, A1 (TF/(TF + TR + TB)), E5 (TF/TR) and F6 (TF/TB) in SSDT by 35.62, 36.6, 37.44 and 36.27%, respectively (*p*<0.05), but obviously increase the levels of TR, B2 (TR/(TF + TR + TB)) and D4 (TR/TB) by 4.86, 3.08 and 4.17%, respectively (*p*>0.05), compared with CK after fermentation (Figure 4A). Moreover, *D. hansenii* Y4 apparently influenced the CIELab parameters of dried tea and tea liquor when it mediated SSDT fermentation, it dramatically increased the levels of BI, Sab, b, Cab, h, a and Hab of dried tea color by 156.37, 54.4, 50, 34.75, 27.78, 16.67 and 14.4%, respectively, while, decreased L level by 12.9%, compared to CK after fermentation (Figure 4B). At the same time, it also significantly enhanced the levels of h and Hab of tea liquor by 12.36 and 6.98% respectively, as compared with CK at the end of fermentation, but obviously decreased the levels of Ps, a, Cab, Eab, and Sab of tea liquor by 58.58, 12.31, 6.27, 5.26, and 4.40%, separately (Figure 4C). It was worth noting that the CK and *D. hansenii* Y4 samples can be clearly distinguished from each other after fermentation based on their dried tea color parameters (OPLS-DA model: R^2^Y = 0.881, Q^2^ = 0.577; cross-validation with 500 permutation tests: intercepts of R^2^ = 0.555, Q^2^ = –0.567), and BI, L, b, Cab and Eab were the differential color parameters between them (Figure. 4D). Moreover, at the end of fermentation, the CK and *D. hansenii* Y4 samples could also be clearly separated from each other based on tea liquor color parameters (OPLS-DA model: R^2^Y = 0.942, Q^2^ = 0.843; cross-validation with 500 permutation tests: intercepts of R^2^ = 0.373, Q^2^ = –1), and BI, Ps, a, Cab and Eab were their differential color parameters (Figure 4E). Therefore, *D. hansenii* Y4 seriously influenced the color parameters of SSDT and exhibited a strong capacity to enhance tea “reddish brown” factors through increasing the levels of BI and TR but decreasing the levels of TF and its derived parameters.

### Expression characteristics of *GH* gene in *Debaryomyces hansenii* Y4 during SSDT fermentation

**Figure 5:**
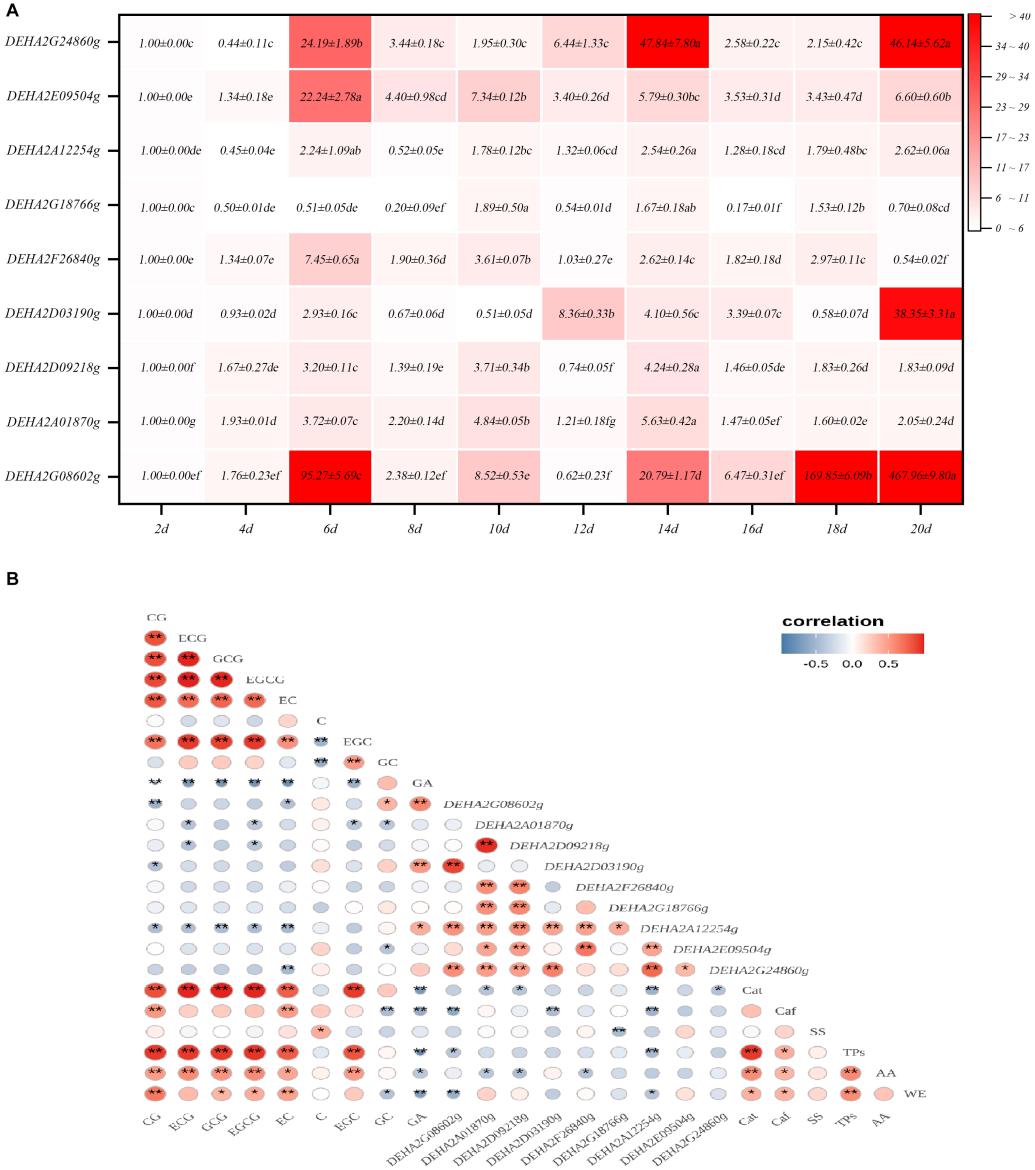
Analysis of *GH* gene expression characteristics in *D. hansenii* Y4 during solid-state fermentation. **(A)** Expression characteristics of *GH* genes; different letters on the gradient color block denote a significant difference at *p* = 0.05. **(B)** Correlation analysis on the *GH* gene expression level and Chemical component level, * and ** reflect a significant difference at *p* = 0.05 and *p* = 0.01, respectively.

Nine *GH* genes were selected for expression analysis. These genes encode GHs in the relatively abundant families and are presumably involved in cell wall degradation. It was found that the expression levels of *GH* genes showed a significant fluctuation with fermentation, of which most genes exhibited a significant up-regulation of expression. Notedly, *DEHA2G24860g* (beta-galactosidase gene) and *DEHA2G08602g* (mannan endo-1,6-alpha-mannosidase DFG5 gene) were dramatically up-regulated in the fermentative anaphase (*p* < 0.05), whose expression levels at 14d were 47.84 and 20.17-fold higher than that at 2d, respectively. Furthermore, at 18d, the expression level of *DEHA2G08602g* was 169.85-fold higher than that at 2d, while at the end of fermentation, the expression levels of *DEHA2G24860g* and *DEHA2G08602g* were 46.14 and 467.96-fold higher than that at 2d. In contrast, the expression level of *DEHA2G18766g* (glucan 1,3-beta-glucosidase gene) was significantly down-regulated during fermentation (*p* < 0.05), while the expression levels of *DEHA2A12254g* (glucoamylase gene) and *DEHA2D09218g* (mannosyl-oligosaccharide 1,2-alpha-mannosidase gene) presented a down-regulation trend during fermentative metaphase and anaphase (Figure 5A). Additionally, the correlation between the expression level of *GH* gene and the content of chemical component was analyzed. It was evident that the expression of most *GH* genes was significantly correlated with the content of catechin monomers. Among them, the expression level of *DEHA2G086*02g was significantly and positively related to GA and GC contents, but negatively correlated to EC, CG, Caf and TPs levels (*p* < 0.05), while *DEHA2D03190*g was significantly and positively related to GA level, but negatively correlated to CG level (*p* < 0.05). Moreover, *DEHA2G24860g* was significantly and negatively related to EC content, while *DEHA2D09218g* and *DEHA2A12254g* were significantly and negatively correlated to ECG and EGCG contents, and *DEHA2A12254g* was also significantly and negatively related to Caf and TPs (*p* < 0.05). It seems that *DEHA2G086*02g is a pivotal functional gene in *D. hansenii* Y4 affecting SSDT quality formation during fermentation.

## Discussion

*Debaryomyces* is beneficial for the development of dark tea flavor during production (Li M. Y. et al., 2020). Usually, *D. hansenii* discovered in protein-rich fermented products exhibits the characteristics of being metabolically versatile, non-pathogenic and tolerant to low temperatures (Viana et al., 2011). However, *D. hansenii* Y4 used in this work was isolated from the high-temperature location of piled SSDT and subjected to high-temperature solid-state fermentation, implying that *D. hansenii* Y4 may be a thermophilic yeast.

Glycoside hydrolases are famous for their excellent capacity to catalyze the hydrolysis of glycosidic linkages (Davies and Henrissat, 1995; Drula et al., 2022). Some GHs identified in *D. hansenii* may strongly participate in the hydrolysis of polysaccharides and oligosaccharides, for example, α-D-galactoside galactohydrolase is mainly responsible for cleaving terminal α-1,6-linked D-galactosyl residues from oligosaccharides substrates, while β-galactosidase could efficiently hydrolyze disaccharide lactose to generate galactose and glucose, and also has the ability to facilitate the transgalactosylation reaction of lactose to allolactose, which is finally cleaved to monosaccharides. Furthermore, both beta-mannosidase and beta-galactosidase are oligosaccharide-degrading enzymes (Mhuantong et al., 2015). It was demonstrated that *D. hansenii* Y4 may mediate the metabolism of polysaccharides or oligosaccharide in tea leaves during fermentation via GHs, then part of the resultant monosaccharides would be used to sustain microbial growth, and the remainder may increase the SS level and finally benefit SSDT sweetness. Additionally, most of the GHs detected were located in the extracellular space, that means they can act directly on the tea leaves to stimulate chemical reactions. Regrettably, the *GH* genes detected in *D. hansenii* Y4 were not as abundant as those found in *Aspergillus* fungi (Ma et al., 2021; Zou et al., 2022, 2023).

In general, the transformation of the chemical component in SSDT during pile-fermentation always results from a synergistic effect of moist heat and microbial activity (Zou et al., 2022), whereas, after excluding the effect of hygrothermal action used the control treatment, apparently, *D. hansenii* Y4 seriously reduced the contents of TPs and Caf, and altered the levels of catechin monomers in tea. Previous literature suggested that GHs, tannase, glycosyltransferases and so on could catalyze the hydrolysis of phenolic glycosides and phenolic esters, oxidation or polymerization of phenolic compounds, and destruction of the aromatic rings in phenolic compounds, thus significantly reducing the TPs levels in tea during fermentation (Ma et al., 2021). Moreover, *D. hansenii* was reported to be a tannin-tolerant yeast with an excellent capacity of secreting tannase (Kanpiengjai et al., 2016), combined with the GHs detected in this work, it was speculated that *D. hansenii* Y4 may be an important functional microbe influencing the conversion of bitterness and astringency-active compounds during SSDT pile-fermentation.

Catechins are the major constituents of TPs, *D. hansenii* Y4 seems to strongly affect the conversion of catechin monomers in this work. It dramatically enhanced ECG level, but decreased CG level in tea after fermentation, implying that this yeast may efficiently facilitate the isomerization reaction between ECG and CG. Unexpectedly, some interesting relationships between the contents of catechin monomers and the expression levels of *GH* genes were detected, for instance, the content of EC was significantly and negatively related to the expression levels of *DEHA2G24860g* and *DEHA2G08602g*, respectively, while the content of CG was significantly and negatively correlated with the expression levels of *DEHA2G08602g* and *DEHA2D03190g*, respectively. In view of the apparent decrease in the levels of EC, EGCG and so forth after fermentation and the relationship between catechin monomers and *GH* genes, it cannot be excluded that the expression of some *GH* genes probably leads to an obvious decrease in the levels of specific catechin monomers by mediating the glycosylation of catechins, as researchers have discovered that certain GHs can catalyze transglycosylation reactions, e.g., transferring a sugar unit to a nucleophilic acceptor other than water under certain conditions (Prieto et al., 2021; Wang et al., 2022). Cho et al. (2011) have employed amylosucrase from *Deinococcus geothermalis* DSM 11300 to biosynthesize (+)-catechin glycosides via linking glucose or maltose molecule to (+)-catechin, and Méndez-Líter et al. (2022) also successfully synthesized 3 novel EGCG-glycosides utilizing engineered transformed β-glucosidase and β-xylosidase from *Talaromyces amestolkiae.* The production of glycosylated catechin will result in an evident reduction in the levels of some unique catechin monomers. Besides, *DEHA2G24860g* and *DEHA2G08602g* were sharply up-regulated in the anaphase of high-temperature fermentation, suggesting that they are likely to be thermotolerant genes.

In this work some functions of *D. hansenii* Y4 during SSDT fermentation were verified, but compared to the functional research of filamentous fungi during dark tea fermentation, which is obviously insufficient (Ma et al., 2021; Zou et al., 2022, 2023; Liao et al., 2023). We expect to explore more functional microbes during SSDT production, and we will continue to investigate the activities of unique GHs in *D. hansenii* Y4 for elucidating its action mechanism during SSDT pile-fermentation and facilitating its application in our subsequent work.

## Conclusion

In this work, many genes encoding GHs, distributed in a comparatively abundant GH17, GH18, GH76, GH31, GH47 and GH2, were detected in *D. hansenii*, and most of the GHs were located in the exocellular space. *D. hansenii* Y4 exhibited an excellent ability to improve the mellow mouthfeel of SSDT via increasing the WE, SS and AA contents, but reducing the TPs and Caf levels. It seriously influenced the “reddish-brown” factors of SSDT and possibly accelerated the isomerization reaction between ECG and CG. *DEHA2G08602g* (mannan endo-1,6-alpha-mannosidase DFG5 gene) in *D. hansenii* Y4 was dramatically up-regulated in fermentative anaphase, and its expression was significantly and negatively correlated to EC and CG levels. Overall, *D. hansenii* Y4 may be an important functional microbe targeting carbohydrates degradation and catechin transformation during SSDT pile-fermentation, with *DEHA2G08602g* as a pivotal thermotolerant *GH* gene. These results may provide a novel complement to the traditional theory of dark tea pile-fermentation.

## Data availability statement

The original contributions presented in the study are included in the article/Supplementary material, further inquiries can be directed to the corresponding author.

## Author contributions

YZ and WX designed the experiments. ML, YuL, XuL, XiL, and YiL performed experiments. YZ and ML wrote the manuscript. QT reviewed and edited the manuscript. All authors contributed to the article and approved the submitted version.

## Funding

This work was supported by the National Natural Science Foundation of China (32102434), Tea Refining and Innovation Key Laboratory of Sichuan Province, and the “Disciplinary Construction Double Support Program” of Sichuan Agricultural University.

## Conflict of interest

The authors declare that the research was conducted in the absence of any commercial or financial relationships that could be construed as a potential conflict of interest.

## Publisher’s note

All claims expressed in this article are solely those of the authors and do not necessarily represent those of their affiliated organizations, or those of the publisher, the editors and the reviewers. Any product that may be evaluated in this article, or claim that may be made by its manufacturer, is not guaranteed or endorsed by the publisher.

## Footnotes

^1^ https://ftp.ncbi.nlm.nih.gov/genomes/

^2^ http://pfam.xfam.org/

^3^ https://web.expasy.org/protparam

^4^ https://software.informer.com/search/MapInspect
