# Supplementary material for: The glycoside hydrolase gene family profile and microbial function of Debaryomyces hansenii Y4 during South-road dark tea fermentation
Source: Front Microbiol. 2023 Jul 12;14:1229251. doi: 10.3389/fmicb.2023.1229251 (PMC10369063; doi:10.3389/fmicb.2023.1229251)
Supplement: Supplementary file 1 [file Table_1.DOCX]

TABLE S1 HMMER3.0 profile of the GHs domain

| No. | GH module |
| --- | --- |
| 1 | ACC PF00703.24 |
| 2 | ACC PF02056.19 |
| 3 | ACC PF00723.24 |
| 4 | ACC PF00722.24 |
| 5 | ACC PF00332.21 |
| 6 | ACC PF00704.31 |
| 7 | ACC PF00182.22 |
| 8 | ACC PF16499.8 |
| 9 | ACC PF01055.29 |
| 10 | ACC PF01532.23 |
| 11 | ACC PF03663.17 |
| 12 | ACC PF03537.16 |
| 13 | ACC PF04041.16 |
