# Supplementary material for: The glycoside hydrolase gene family profile and microbial function of Debaryomyces hansenii Y4 during South-road dark tea fermentation
Source: Front Microbiol. 2023 Jul 12;14:1229251. doi: 10.3389/fmicb.2023.1229251 (PMC10369063; doi:10.3389/fmicb.2023.1229251)
Supplement: Supplementary file 2 [file Table_2.DOCX]

TABLE S2 Formulas for calculating the derived color parameters

| Item | Formulas |
| --- | --- |
| Derive parameters of tea pigments | A1=TF/(TF + TR + TB) |
|  | B2=TR/(TF + TR + TB) |
|  | C3=TB/(TF + TR + TB) |
|  | D4=TR/TB |
|  | E5=TF/TR |
|  | F6=TF/TB |
|  | G7=(TF + TR)/TB |
|  | H8= (TF + TR)/(TF + TR + TB) |
| Derive parameters of CIELab parameters | Eab=(L^*2^+a^*2^+b^*2^)^1/2^ |
|  | Cab=(a^*2^+b^*2^)^1/2^ |
|  | Sab=Cab/L^*^ |
|  | Hab=tan^-1^(b^*^/a^*^) |
|  | h=b^*^/a^*^ |
|  | Ps=b^*^/(b^*^-a^*^) |
|  | BI=[100(X-0.31)/0.17], X=(a^*^+1.75L^*^)/(5.645L ^*^+a^*^-3.012b^*^) |
