# Supplementary material for: The glycoside hydrolase gene family profile and microbial function of Debaryomyces hansenii Y4 during South-road dark tea fermentation
Source: Front Microbiol. 2023 Jul 12;14:1229251. doi: 10.3389/fmicb.2023.1229251 (PMC10369063; doi:10.3389/fmicb.2023.1229251)
Supplement: Supplementary file 3 [file Table_3.DOCX]

TABLE S3 Primer sequence

| Gene name | Description |  | Primer sequence | GH family |
| --- | --- | --- | --- | --- |
| *DEHA2G24860g* | Beta-galactosidase | F | TTCCTGGTCATTGGCAACTACAAGG | GH2 |
|  |  | R | GGTTCTGCGATGGTGGATTAGGAG |  |
| *DEHA2E09504g* | Mannosidase | F | GGACTTCTCGGGGACCTTGGAG | GH2 |
|  |  | R | TTCGGTAGTTTGGTTGGGTGCTAAC |  |
| *DEHA2A12254g* | Glucoamylase | F | AGAAGGAACGGAGGTTAGTGAAGGG | GH15 |
|  |  | R | GCAGCATCTCTGACCCATTGGTAG |  |
| *DEHA2G18766g* | Glucan 1,3-beta-glucosidase | F | GAAGATTTGACTGCCGACGAATTGG | GH17 |
|  |  | R | ACCGTTCTTGTCCTTGATGTCCTTC |  |
| *DEHA2F26840g* | Alpha-D-galactoside galactohydrolase | F | ACCGCTGTCCTTGTGAATCTTACG | GH27 |
|  |  | R | TCGTTCCATCCCTGACCTGTTCC |  |
| *DEHA2D03190g* | Glucoamylase | F | GGAGAGCCTTGAGTGGAGTTGTTG | GH31 |
|  |  | R | GCTGGCAAACCGATTTCGTGAAC |  |
| *DEHA2D09218g* | Mannosyl-oligosaccharide 1,2-alpha-mannosidase | F | GTTGCCTTATGCTTCCGTGAACTTG | GH47 |
|  |  | R | TGTTGCCACTTCTGCTGTAGATGAC |  |
| *DEHA2A01870g* | Mannan endo-1,6-alpha-mannosidase DCW1 | F | GTGATGGCAGCGACCGAGAAG | GH76 |
|  |  | R | AACCCTCCGTGGCATTCTTTGTG |  |
| *DEHA2G08602g* | Mannan endo-1,6-alpha-mannosidase DFG5 | F | AATATCGCTGCTCGTTTGGCTAGG | GH76 |
|  |  | R | AACCCAACTTCTTCCATCCAATCCC |  |
| 26S | 26S ribosomal RNA | F | GCATATCAATAAGCGGAGGAAAAG |  |
|  |  | R | GGTCCGTGTTTCAAGACGG |  |
