# Supplementary material for: The glycoside hydrolase gene family profile and microbial function of Debaryomyces hansenii Y4 during South-road dark tea fermentation
Source: Front Microbiol. 2023 Jul 12;14:1229251. doi: 10.3389/fmicb.2023.1229251 (PMC10369063; doi:10.3389/fmicb.2023.1229251)
Supplement: Supplementary file 4 [file Table_4.docx]

TABLE S4 Putative conserved motifs information of GHs in *Debaryomyces hansenii*

| Name | E-value | width | Best possible match |
| --- | --- | --- | --- |
| Motif 1 | 1.20E-26 | 41 | YPQTPMYJKMGLWAGGDSSNEPGTIEWAGGETDYSELPFSM |
| Motif 2 | 9.50E-19 | 49 | AKRFDNPAJSSSFYIMYGKVEVKLKAAHGSGIISSFYLQSDDLDEIDIE |
| Motif 3 | 2.50E-16 | 50 | FNTMDARWDHEECGGGLRWQIFQWNSGYDYKNSIANGCLFNIGARLARFT |
| Motif 4 | 3.60E-14 | 29 | PLNNFHTYTIDWTEDKIVWYLDGEVVRTL |
| Motif 5 | 6.70E-10 | 50 | RSAFAGSQKHAAHWGGDNTSDWEWMKFSIPQALSLGLSGIPFWGVDVGGF |
| Motif 6 | 2.00E-09 | 50 | ELRSLFDKDSSKSYYLSAAPQCPYPDZSVGDLLSEVDLDFAFIQFYNNYC |
| Motif 7 | 3.20E-09 | 20 | FQSNYFSKGDTTTYDRGEYH |
| Motif 8 | 1.90E-07 | 27 | WHTCGJBWRNGGWDGFYGLGEQMCALE |
| Motif 9 | 3.70E-06 | 27 | REKFATNVVNFLKKYNLDGIDLDWEYP |
| Motif 10 | 1.40E-05 | 50 | HCSQIGEDIKTCQDNGKIVLLSLGGGIGNYGFDSDAEGQDFAKTLWNKFG |
| Motif 11 | 8.50E-05 | 49 | NVAVYWGQNSGGGQDRLSTYCESDAVDIVLLSFLNNFPGDFNJDFANQC |
| Motif 12 | 2.10E-04 | 37 | YIIYGPDPKDIJQRYAQJTGLPALPPYWALGYHQCRW |
| Motif 13 | 4.30E-04 | 48 | NCTDITDKKWSYTAGIALSGCAYLYNYTEDSIWKDRAIEJAKAAINFF |
| Motif 14 | 6.90E-04 | 41 | HQAGEDFNYIPLNQSMVEGNDDQGFWGLAVMEAVEKNFSDP |
| Motif 15 | 2.70E-03 | 44 | NNPGLCDISDYLAVNAHPFWDGGVVPDNAGSWLLQQIQRLESAC |
