# Supplementary figures and images for: The glycoside hydrolase gene family profile and microbial function of Debaryomyces hansenii Y4 during South-road dark tea fermentation
Source: Front Microbiol. 2023 Jul 12;14:1229251. doi: 10.3389/fmicb.2023.1229251 (PMC10369063; doi:10.3389/fmicb.2023.1229251)

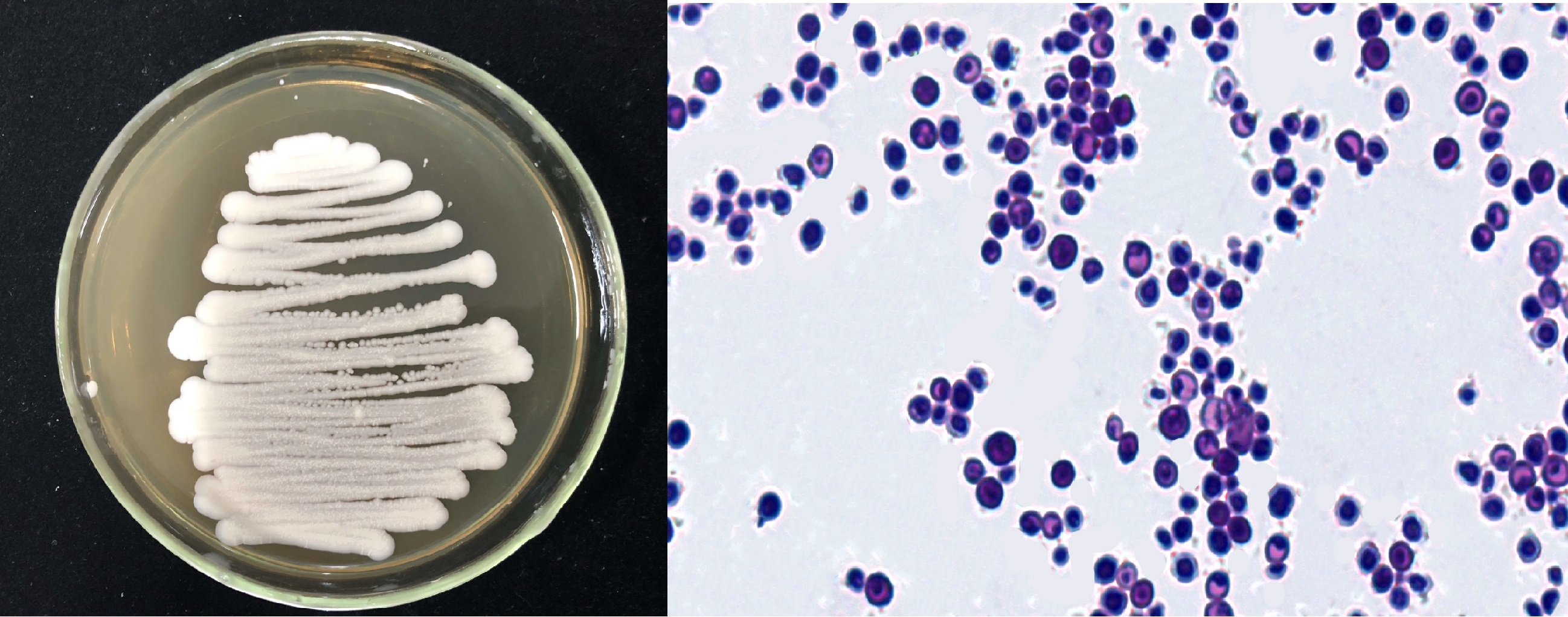

Supplement: Supplementary file 5 [file Image_1.JPEG]
